# Book Notices, Etc.

**Published:** 1866-08

**Authors:** 


### EDITORIAL.

### BOOK NOTICES.

*Researches on the Medical Properties and Application of Nitrous Oxide, etc.* By G. J. ZIEGLER, M. D., Physician to the Philadelphia Hospital, etc.

*Instruction in the Preparation, Administration and Properties of Nitrous Oxide, etc.* By G. T. BARKER, D. D. S., Professor of the Principles of Dental Surgery, etc., in the Pennsylvania College of Dental Surgery, etc.

It undoubtedly remains for the dental profession to present a practical solution of the problem how to secure the simplest and most convenient means of producing and administering nitrous oxide gas as an anæsthetic agent.

It can scarcely be expected that this gas, unless the apparatus for its preparation is much improved, can take the place of ether and chloroform in the ordinary cases for which anæsthetics are required by the surgeon and physician in general practice. For this purpose, the apparatus at present is too bulky and inconvenient for transportation from one patient to another.

With the dentist, however, the case is somewhat different. The operations he is called upon to perform are numerous, painful and usually of short duration, and performed at his office. It is therefore desirable that he should possess an agent which acts speedily, safely, and does not necessitate a long detention of his patient on account of nausea or semi-unconsciousness.

It would seem that protoxide of nitrogen is the most convenient anæsthetic known for the dentist, since it possesses the desirable properties just mentioned, and can be readily administered by means of tubing leading from the apparatus.

For the same reasons this agent may possibly supercede ether and chloroform in hospital practice.

The little volumes of Dr. Ziegler and Dr. Barker present concise statements of the most important practical points regarding the preparation and administration of this gas. This whole subject is an important one, and merits the careful attention of the profession.

It should not be forgotten that to dentists belong the honor of first practically demonstrating the safety of the use of anæsthetics, and, to a certain extent, of introducing them into general use. This fact, in a measure, confers an honor upon the whole dental profession, for which it may well be proud.

We cannot leave this subject without reminding our readers of a fact they can scarcely have overlooked, that the dentists of this country are securing for their profession a high position, not only as an art but as a science.

The art is carried to a perfection, which may excite the wonder of all who examine with care the difficulties which are overcome in the construction of artificial teeth and palates, and in preventing the loss of the natural teeth by different modes of filling.

The energy and success with which so many members of the dental profession are devoting themselves to the investigation of all that pertains to the anatomy, physiology and diseases of the mouth; the skill which they manifest in the treatment of diseases of the jaw and gums, in supplying the deficiency of cleft palate and the management of broken jaw, must command the respect of all educated physicians.

The character of the works on dental surgery, of the periodicals devoted to the interests of the dental profession, the number and tone of the various associations of dentists, and above all, the growing tendency to require dental students to pursue a course of medical study, are but evidence of the efforts our friends are making to elevate the position of their profession.

This field of labor is a most useful one—the relief of pain, and in no small degree the prevention of serious diseases, not only of the teeth but also of the whole system. The extent to which the dentists of our country educate themselves in everything that pertains to their department, will determine the place they will occupy in relation to the learned professions.

---

*Eighth Annual Report of the Chicago Charitable Eye and Ear Infirmary*, 16 *East Pearson Street, presented by the Board of Surgeons, for the year ending May* 1, 1866.

In accordance with the Constitution and By-Laws of the Association, the Surgeons would most respectfully report:

That during the year ending May 1st, 1866, five hundred and sixteen* patients have been under treatment, making an aggregate of two thousand six hundred and forty-two that have been treated since the opening of the Infirmary in 1858.

The following is a classified list of the diseases which have been treated during the past year:

DISEASES OF THE EYE.

| | | | |
|---|---|---|---|
| Wounds and Injuries | 14 | Diseases of Lachrymal Apparatus | 11 |
| Conjunctivitis, catarrhal | 41 | " Retina and Optic Nerve | 29 |
| " granular | 91 | " Choroid and Sclerotic | 6 |
| " " with vascular cornea | 61 | Opacity of Vitreous Humor | 3 |
| Conjunctivitis neonatorum | 4 | Glaucoma | 1 |
| " purulent | 10 | Diseases of Lens — Cataract and Injuries | 11 |
| " phlyctenular | 20 | Diseases of the Muscles | 3 |
| Diseases of the Cornea | 66 | Abnormal Accommodation | 6 |
| Foreign bodies on Cornea | 7 | Unclassified | 25 |
| Diseases of Lids | 33 | | |
| " Iris | 9 | Total | 451 |

* In the Report of the Committee of the Illinois State Medical Society on Diseases of the Eye, page 301 of the last issue of the Journal. instead of two hundred and sixteen, read five hundred and sixteen as the number of poor patients who had been treated during the past year at the Infirmary.

DISEASES OF THE EAR.

| | | | |
|---|---|---|---|
| Diseases of the External Meatus... | 20 | Foreign Substances in Ear. ........ | 4 |
| " " Membrana Tympani.................................... | 8 | Diseases of Mastoid Processes...... | 1 |
| Diseases of the Auditory Nerve.... | 9 | Polypus.................................... | 1 |
| Impacted Cerumen ..................... | 5 | Unclassified............................. | 6 |
| Catarrhal Deafness.. ................. | 11 | | 65 |

The Surgeons take pleasure in stating that a larger number of poor patients have sought advice and treatment at the Infirmary during the past than any previous year. It is a proof of the continued confidence with which its efforts are elsewhere regarded, that the expenses of patients from a distance have, not unfrequently, been borne by benevolent individuals in their respective communities. Physicians also in various parts of the Northwest have expressed warm interest in the welfare of this charity, and have frequently testified to the great good it has accomplished.

The benefits which many of these patients have received can scarcely be estimated. Parents, unfortunate and helpless, have been restored to sight and the means of supporting their families. A large number of children have been rescued, it is believed, from partial or total blindness—and thus from life-long poverty. Many patients, affected with diseases, trifling in themselves, but which, if neglected, are almost certain to result in permanent injury of vision, have been relieved by short and simple treatment. And it is upon this class of patients, that the Surgeons look with peculiar interest, as illustrating the manner in which this charity, if possible, accomplishes most good, by *encouraging poor patients, without fear of expense, to seek medical aid in the very commencement of their diseases, before they have assumed a dangerous form.*

There is scarcely any form of charity, whose claims can be so forcibly urged on the grounds of humanity and economy, as this. It relieves physical suffering and mental distress by the cure of painful diseases, and by removing fears of threatened blindness; it restores many with impaired vision to sight, and to their daily labors, thereby removing one cause of poverty; it prevents ignorance by rescuing poor children from partial or total loss of sight—thus enabling them to acquire the rudiments of knowledge and to follow in after life honorable and remunerative occupations. How much more in accordance with an enlightened humanity is it to relieve such children, than to neglect them till they become dependent upon the Blind Asylum for their education and limited means of support.

On the ground of economy this charity claims *especial* consideration. So far as it prevents blindness—so far it lessens taxation by reducing the number of the poor dependent upon public or private charity—and so far it adds to the productive labor and wealth of the State. Its principle is Prevention, one of the first laws of Economy. It does not encourage the habit of idleness and begging, as, it is feared, is too often the case with direct pecuniary aid. It restores to health, hope and useful activity. It would be difficult to point to another form of charity by which so much good can be accomplished at so little cost.

During the war of the Rebellion, a large number of soldiers, soldiers' wives and children received gratuitous treatment at the Dispensary of the Infirmary for diseases of the eye or ear.

The Infirmary has been able during the past year to extend its charities to soldiers more largely than before, in consequence of the action of the N. W. Sanitary and Christian Commissions.

In view of the number of discharged soldiers with diseases of the eye, who were constantly applying for medical and surgical aid, the Northwestern Branch of the U. S. Sanitary Commission not only contributed valuable supplies of household furniture, especially beds, bedding and clothing, but also, by recommendation of the U. S. Sanitary Commission, created a fund for the support of soldiers at the Infirmary while under treatment.

The N. W. Sanitary Commission, also by recommendation of the U. S. Sanitary Commission, donated $500 to aid in constructing the enlargement of the Infirmary building, rendered necessary by the number of patients thus increased by soldiers seeking treatment.

The N. W. Christian Commission also donated the sum of $500 for the same purpose.

The number of soldiers in Illinois and the whole Northwest, who are incapacitated for self-support by diseases of the eye, contracted in the army, and, for this reason, thrown upon the charities of their friends or the public, is known to be very large. For a long time this class of patients must depend in a measure for treatment upon such Institutions as this.

The fund created by the Sanitary Commission will enable the Infirmary to support but a limited number of the soldiers who will need this form of charity. Will not the public contribute means sufficient to spare the Infirmary the necessity of turning from its doors a single soldier deserving the aid it seeks to give?

The Surgeons take pleasure also in stating that the Infirmary was incorporated in February, 1865, by a special act of the General Assembly of the State of Illinois.

This charity is intended for the poor of the Northwest, as well as of Chicago. For such patients, treatment, medicines and lodging will be provided gratuitously. A charge is necessary at present for board, since the means of the Infirmary are insufficient to furnish this last without charge. Efforts will be made to establish a fund by which the poor may also obtain gratuitous board.

The Infirmary now possesses, with its recent enlargement, a commodious hospital building in a healthy portion of the city, near the lake, with all the conveniences necessary for the comfort and welfare of patients suffering from diseases of the eye or ear.

But with its increased capacity come increased calls from the poor for medical aid, and a greater demand on the public to assist the Infirmary in its benevolent labors.

The Infirmary is at No. 16 East Pearson Street, half a mile north of State Street Bridge, and is open at all times for the reception of the poor, afflicted with diseases of the eye or ear. The Dispensary of the Infirmary is also open from 2 to 2½ P.M. for such poor patients, not boarding at the Infirmary, as may require treatment.

---

WANTED, by Dr. J. H. Wilson, Marion, Linn county, Iowa, Nos. 1 and 6, 1864, and No. 7, 1865, of the Journal, for which a handsome price will be paid.

*Why Not? A Book for Every Woman. The Prize Essay to which the American Medical Association Awarded the Gold Medal for* 1865. By H. R. Storer, M. D., of Boston. Lee & Shepard, Boston.

In spite of the unfortunate title of this little volume, and in spite of certain peculiarities which lay it open to severe criticism, were any one sufficiently captious to quarrel with a work which conveys the truths which this contains, we are glad to see that Dr. Storer's tract is at length before the public.

Total ignorance of all that the natural instinct does not impart concerning the sexual functions, would be safer and better than the fancied knowledge with which the men and women of the present day have been led to suppose themselves in advance of their simple-minded ancestors. But since the innocent days of the childhood of the world in such matters cannot be recalled, the only safety of the race is to be found in complete information. This it is the duty of our profession to convey. We therefore cordially commend this essay to the attention of all for whom it was designed, believing that a flood of suffering and wretchedness may thus be averted from many a family throughout the land.

For sale by Cobb & Pritchard, Lake street, Chicago.

---

We glean from the *American Literary Gazette* the following lists of books, either recently published or in course of preparation for the market:

*List of Medical Books recently published in the United States.*

Bennett's Clinical Lectures. *Third Edition.*
Da Costa's Medical Diagnosis. *Second Edition.*
Echeverria's Reflex Paralysis.
Schmoele on Asiatic Cholera.
Seguin on Idiocy and its Treatment by the Physiological Method.
Sim's Clinical Notes on Uterine Surgery.
Storer's Why Not? A Book for Every Woman.

*Works announced for early publication.*

Aitken's Science and Practice of Medicine. From the Fourth London Edition.
Basham on Dropsy.
Beale on the Microscope in Practical Medicine.
Dixon's Treatise on Diseases of the Eye.
Duchenne's Localized Electrization.
Ellis on the Safe Abolition of Pain in Labor and Surgical Operations.
Prince's Orthopedic Surgery.
Remak's Electro-Therapeutics.
Reynolds on Diseases of the Brain and Nervous System.
Trousseau's Clinical Medicine. Vol. I, Part I.
Waring's Practical Therapeutics.
Zander on the Ophthalmoscope.

*Books recently published in the United States, but manufactured abroad.*

Beale's How to Work with the Microscope.
Beraud's Atlas of Surgical and Topographical Anatomy.
Holmes' System of Surgery.

*Works recently published in Great Britain.*

Annandale's Surgical Appliances and Operative Surgery.
Barclay's Gout and Rheumatism.
Barker's Diseases of the Respiratory Passages.
Griffith's Dermatology. Treatment of Skin Diseases.
Leach's Brief Notes on the Cholera in Turkey.
Macpherson's Cholera in its Home.
Moon's Handy-Book of Ophthalmic Surgery.
Smith's Common Nature of Epidemics.

---

OBITUARY.—PETER T. LANGE, M. D., Chicago, drowned while bathing in the lake, July 14, 1866. The deceased was a member of the last class which graduated from Rush Medical College, and had engaged in the practice of his profession in this city with the fairest prospects of briliiant success.

